# Intraoperative closure of a large colonic perforation using dual adjustable instrument and channel endoscopy

**DOI:** 10.1055/a-2621-3297

**Published:** 2025-07-02

**Authors:** Kazuya Sumi, Yuki Kawasaki, Hisaki Kato, Taro Tanabe, Masayuki Isozaki, Noboru Yokoyama, Haruhiro Inoue

**Affiliations:** 1 378609 Digestive Diseases Center, Showa University Koto Toyosu Hospital, Tokyo, Japan

Various techniques have been reported for closing perforations and defects following endoscopic procedures 1 2 3 4 5 , with dual-channel endoscopy being one of them. However, its availability is limited, and the fixed orientation of the forceps channel restricts its flexibility. Dual adjustable instrument and channel endoscopy (DAICE) is a novel approach for closing large defects. DAICE converts the standard endoscope into an adjustable dual-channel system with an external channel hood (ECH; Top Corporation, Tokyo, Japan) aligned to the defect.

A woman in her 60s who had undergone the Hartmann’s procedure for sigmoid colon diverticular perforation was admitted for colostomy closure and anastomosis. A full-thickness upper rectal tear on the mesenteric side was noted during transanal anastomosis ( Fig. 1 ). Given the anatomical location, direct surgical closure was deemed technically unfeasible ( Fig. 2 ). Consequently, intraoperative endoscopic closure was planned.

**Fig. 1:**
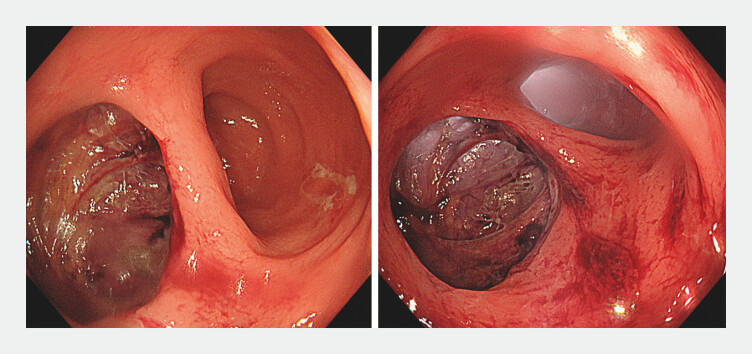
A defect comparable in size to the colon lumen was noted.

**Fig. 2:**
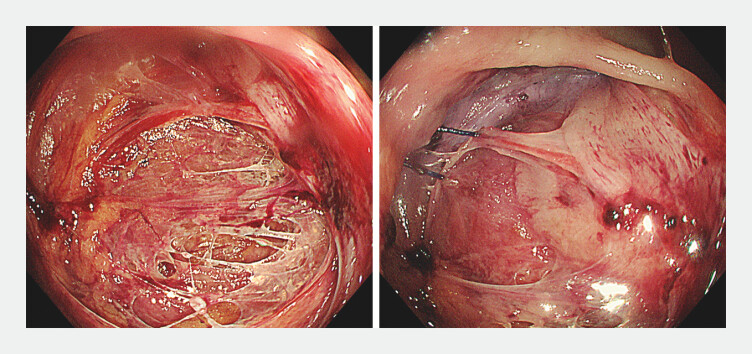
Surgical closure was attempted but ultimately could not be performed. The sutures used during the procedure were found.

Closure with an over-the-scope clip was not feasible owing to the large defect size and the fixed channel orientation hindered proper clip engagement on the deeper edge of the defect. To overcome this, DAICE was developed by attaching an ECH to the standard endoscope, allowing positional adjustment. One side of the defect was grasped using a rat tooth forceps and brought closer to the opposite side. The clips were inserted through the external channel, starting from the posterior edge, to close the defect ( Fig. 3 , Fig. 4 ).

**Fig. 3:**
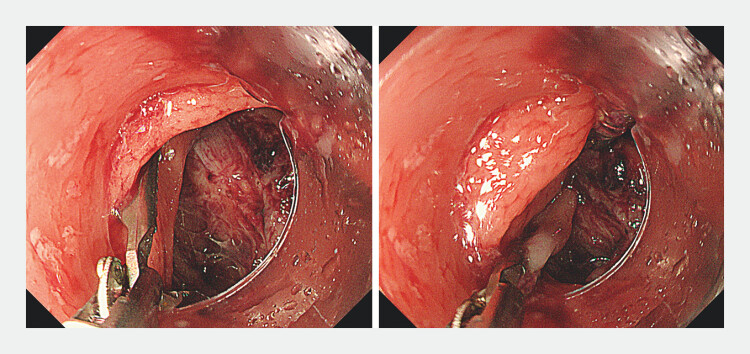
An external channel hood was applied to perform dual adjustable instrument and channel endoscopy. One side of the defect was grasped with forceps and brought toward the opposite side, allowing approximation and closure of the defect using clips.

**Fig. 4:**
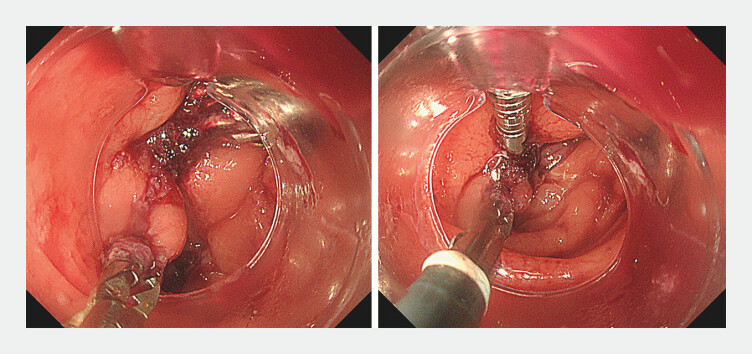
The position of the external channel hood was adjusted to align with the orientation of the closure.

The procedure was completed after confirming no air leaks and complete closure without luminal stenosis ( Fig. 5 , Video 1 ).

**Fig. 5:**
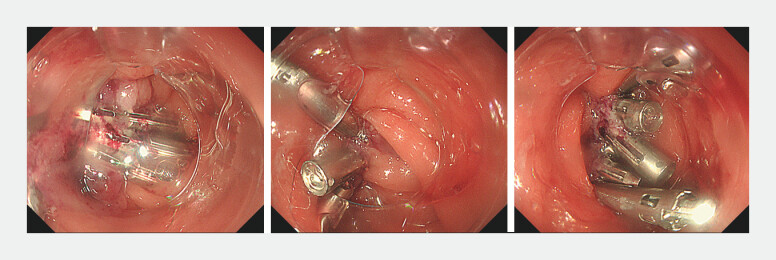
The large perforation was successfully closed by dual adjustable instrument and channel endoscopy.

Using an external channel hood and adjusting it to align with the closure (dual adjustable instrument and channel endoscopy), the large surgical perforation was successfully closed endoscopically.Video 1

DAICE is effective for closure, and is versatile and applicable in various scenarios. The 2.8-mm guide tube of the external channel is compatible with a range of devices, enabling its various clinical applications. The ECH can be attached easily, and the procedure can be performed with conventional endoscopes, eliminating the need for dual-channel endoscopy, thereby reducing cost.

Endoscopy_UCTN_Code_CPL_1AJ_2AG

## Notes

Endoscopy E-Videos https://eref.thieme.de/e-videos
*E-Videos* is an open access online section of the journal *Endoscopy* , reporting on interesting cases and new techniques in gastroenterological endoscopy. All papers include a high-quality video and are published with a Creative Commons CC-BY license. Endoscopy E-Videos qualify for HINARI discounts and waivers and eligibility is automatically checked during the submission process. We grant 100% waivers to articles whose corresponding authors are based in Group A countries and 50% waivers to those who are based in Group B countries as classified by Research4Life (see: https://www.research4life.org/access/eligibility/ ). This section has its own submission website at https://mc.manuscriptcentral.com/e-videos .
